# Case report: Atypical teratoid/rhabdoid tumor of the lateral ventricle in a male adolescent (case-based review and diagnostic challenges in developing countries)

**DOI:** 10.3389/fonc.2022.985862

**Published:** 2022-10-06

**Authors:** Akzhol Karim, Kundyz Shaikhyzada, Assel Suleimenova, Bakytkali Ibraimov, Dair Nurgaliev, Dimitri Poddighe

**Affiliations:** ^1^ Clinical Academic Department of Pediatrics, Pediatric Oncology Section, National Research Center for Maternal and Child Health, University Medical Center (UMC), Nur-Sultan, Kazakhstan; ^2^ Clinical Academic Department of Laboratory Medicine, Pathology Section, University Medical Center (UMC), Nur-Sultan, Kazakhstan; ^3^ Department of Medicine, Nazarbayev University School of Medicine, Nur-Sultan, Kazakhstan

**Keywords:** atypical teratoid/rhabdoid tumor, lateral ventricle, pediatric brain tumor, adolescent, case report, case-based review

## Abstract

Atypical teratoid/rhabdoid tumor (AT/RT) is a rare and highly malignant central nervous system (CNS) embryonal neoplasm: it accounts for <2% of all pediatric CNS tumors and occurs mainly in infants and young children. The primary site of this tumor is usually the posterior cranial fossa. Supratentorial and, in detail, latero-ventricular location is extremely uncommon, especially in adolescents. This tumor is characterized by rapid growth and spread in cerebrospinal fluid and, therefore, it is characterized by a poor prognosis. Neurological signs and symptoms are related the location of the tumor. The radiological features of AT/RT are nonspecific. Immunohistochemical staining for loss of nuclear integrase interactor 1 (INI1) expression is considered a reliable criterion for the diagnosis of this type of tumor. AT/RT has been linked to mutations of SMARCB1 or, rarely, SMARCA4 genes, which function as tumor suppressor genes. Currently, there is no validated protocol of treatment for children with AT/RT, and multimodality treatment (consisting of surgery, chemotherapy, and radiation therapy) is considered. In this case report, we describe a 15-year-old adolescent with an AT/RT of the left lateral ventricle. Despite the late diagnosis, the multimodal therapeutic approach provided a good outcome for our patient at 21 months’ follow-up. Based on our case-based review, early diagnosis and a multimodal approach to treatment play a key role in improving the survival of patients with this diagnosis. Implementing a system supporting pathological and molecular analyses for developing countries and, in general, for non-academic centers is of primary importance to timely diagnose and treat rare tumors, such as AT/RT.

## Introduction

Atypical teratoid/rhabdoid tumor (AT/RT) is a highly malignant embryonal neoplasm of the central nervous system (CNS). AT/RT was first described in 1987 and, after it was distinguished from primitive neuroectodermal tumors, it was introduced in the nomenclature of the World Health Organization classification of CNS tumors and International Classification of Diseases for Oncology (third edition) in 2000 (1, 2).

AT/RT is most commonly seen in children under three years of age. AT/RT is predominantly seen in males, with a male-to-female ratio of 2.7:1 (3, 4). This tumor is characterized by rapid growth and spread in cerebrospinal fluid; therefore, it is characterized by a poor prognosis (5, 6). Most cases of AT/RT arise from the posterior fossa or, much less frequently, from the spinal cord; the occurrence of AT/RT in the lateral ventricle is extremely rare in children (6, 7).

Due to AT/RT rapid growth and spreading, these patients usually have a relatively short (in the order of weeks, or even days) clinical presentation characterized by the onset of progressive symptoms. Neurological signs and symptoms are related the site of the tumor. The diagnostic work-up includes CNS magnetic resonance imaging (MRI) and cerebrospinal fluid (CSF) examination; however, AT/RT cannot be reliably distinguished from other malignant brain tumors without histopathological examination: therefore, surgery is necessary to obtain tissue and confirm the diagnosis. AT/RT has been linked to mutations of SMARCB1 or rarely SMARCA4 genes, which function as tumor suppressor genes (7).

Currently, there is no validated protocol of treatment fo children with AT/RT, and multimodality treatment (consisting of surgery, chemotherapy, and radiation therapy) is usually considered.

Herein, we reported a case of AT/RT developing from the lateral ventricle (which is an unusual site for this rare tumor) in an adolescent. Moreover, we performed a case-based review and discussed the main clinical aspects and diagnostic challenges.

## Case description

### Clinical and radiological course

A previously healthy 15 years old boy presented to the regional hospital with chief complaint of headache, which was associated with nausea and emesis. These symptoms gradually worsened, and visual deterioration, strabismus, and unstable gait also appeared within 1 month. The detailed and chronological clinical course is summarized in **Table 1**.

**Table 1:** Timeline of the clinical episode.

September, 2020	Onset of first symptoms (nausea, vertigo and, later, visual impairment)
October 20^th^, 2020	Brain MRI (tumor mass: 6.8 x 4.4 x 5 cm)
November 2^nd^, 2020	Left frontoparietal craniotomy (partial tumor resection)
November 4^th^, 2020	Brain MRI (residual mass:1.3 x 1.1 x 1 cm)
November 17^th^, 2020	Initial diagnosis of germ cell tumor (post-operative adjuvant chemotherapy: SIOP CNS GCT II protocol)
January 25^th^, 2021	Brain MRI (residual mass: 1.8 x 1 cm)
February 2^nd^, 2021	Completion of 4 chemotherapy cycles (according to SIOP CNS GCT II protocol)
March 3^rd^, 2021	Diagnostic revision: AT/RT
March 15^th^, 2021	Start of post-operative adjuvant radiotherapy (1^st^ session)
April 16^th^, 2021	Completion of adjuvant radiotherapy (27^th^ session)
May 25^th^, 2021	Brain MRI (residual mass: 4.5 x 5 mm)
September 7^th^, 2021	Brain MRI (residual mass: 4.5 x 5 mm and 5.6 x 5.2 mm)
December 20^th^, 2021	Brain MRI (residual mass: 4.5 x 4.6 mm and 5.6 x 5.2 mm)
February 3^rd^, 2022	Completion of post-operative adjuvant chemotherapy (14^th^ cycle)
March 30^th^, 2022	Brain MRI: (residual mass: 4.5 x 3.2 mm and 4.3 x 2.6 mm)

MRI, magnetic resonance imaging; AT/RT, atypical teratoid/rhabdoid tumor.

We first assessed this patient at our pediatric tertiary center where he was transferred 15 days after a neurosurgical intervention, which consisted of a left frontoparietal craniotomy. The intra-operative findings revealed a pale and gray mass, characterized by soft consistency and abundant vascularity.

When he arrived at our center, he was conscious, but several neurological alterations were present: pupils were isochoric and reactive, but he showed exotropia of the left eye and bitemporal hemianopsia; moreover, he presented positive Romberg sign. However, muscle tone and deep tendon reflexes were maintained. No other relevant neurological deficits were noticed.

**Figure 1:**
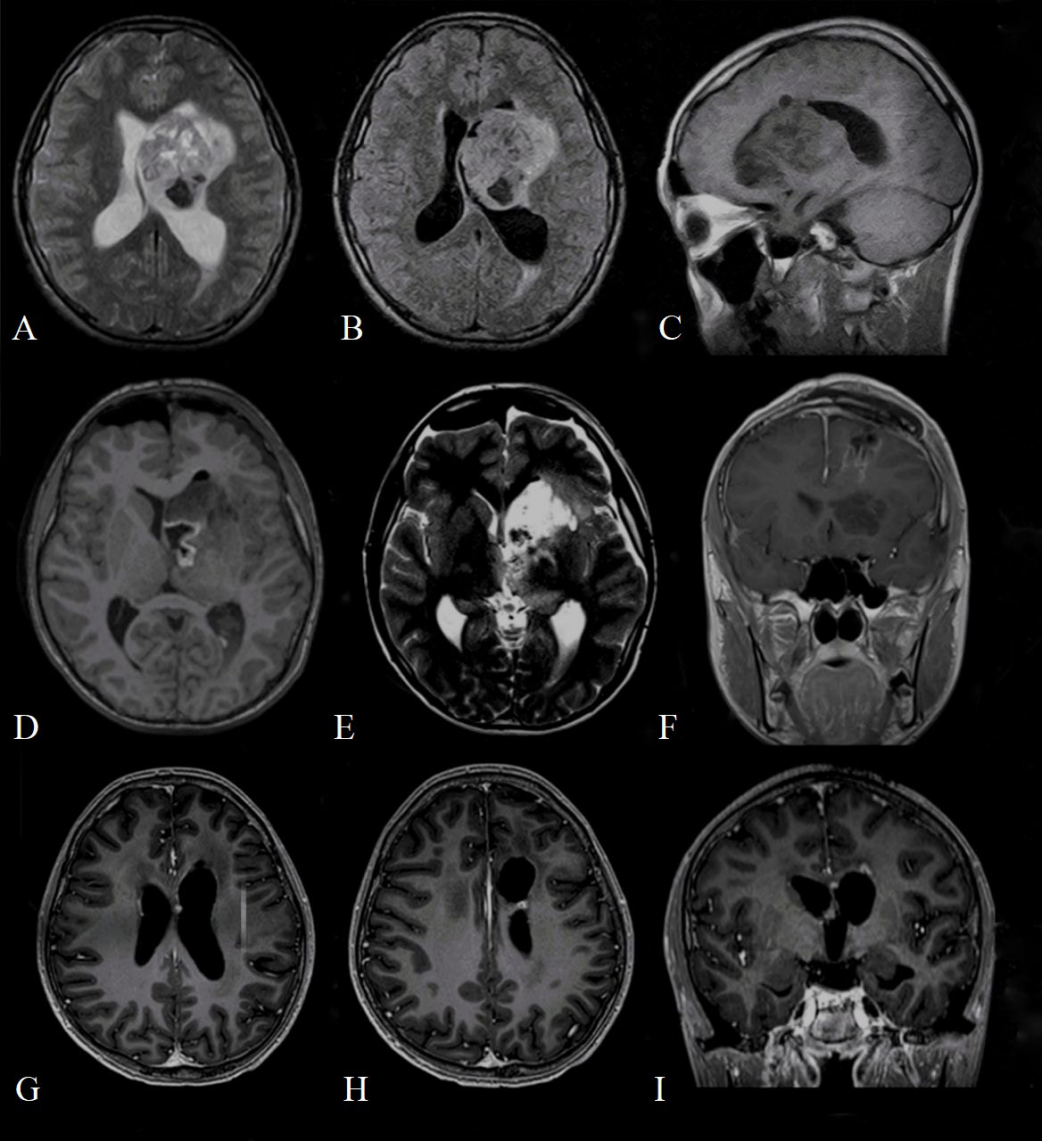
Preoperative **(A)** T2 axial magnetic resonance imaging (MRI); **(B)** T2 FLAIR axial MRI; **(C)** T2 sagittal MRI demonstrating mass in the left lateral ventricle with heterogeneous enhancement measuring 7.2 cm x 4.7 x 5 cm, composed of both cystic and solid areas, and extending to the frontal lobe of the brain; Postoperative **(D)** T1 axial magnetic resonance imaging (MRI); **(E)** T2 weighted image axial MRI; **(F)** T1 contrasted coronal MRI demonstrates the area of postoperative cystic change and residual tumor measuring 1.3 cm x 1.1 x 1 cm, actively accumulating contrast agent; contrasted T1 axial **(G, H)** and coronal **(I)** MRI at 17-month follow-up, which shows residual tumor measuring 4.5 x 3.2 mm **(H)** and 4.3 x 2.6 mm **(G)**.

The first cranial MRI (before neurosurgery) reported a contrast-enhanced mass in the left periventricular region with involvement of the left basal ganglia, head of the caudate nucleus, thalamus, until the frontal lobe and body of the lateral ventricle (**Figures 1A–C**). The lesion (6.8 x 4.4 x 5 cm) consisted of a cystic/solid formation with heterogeneous structure due to areas of hemorrhage; it was hypointense on T1-weighted images, hyperintense on T2-weighted images; it showed irregular borders and was surrounded by a rim of edema. The mass compressed the left lateral and third ventricles, and the midline structures of the brain were displaced to the right, indicating asymmetric internal hydrocephalus. Spinal cord imaging did not show any changes.

The computerized tomography (CT) obtained in the immediate postoperative period showed foci of air and small areas of hemorrhagic component along the periphery of the surgical bed. Brain MRI performed on the 2^nd^ day after surgery showed a residual tumor measuring 1.3 x 1.1 x 1 cm in the region of the left lateral ventricle (**Figures 1D–F**).

During the post-operative neurological examination, there was a regression of neurological symptoms, which was probably associated with a decrease in intracranial pressure and elimination of the mass effect on nearby brain structures. A lumbar puncture was also performed: clear cerebrospinal fluid(CSF) without tumor cells was reported. The levels of alpha-fetoprotein and beta-human chorionic gonadotropin in blood and CSF were within the normal range both before and after surgery.

MRI of the spinal cord performed 3 weeks after neurosurgery did not reveal any signs of metastatic disease. In addition, chest CT and abdominal ultrasound (requested according to the indications of the national medical protocols for patients diagnosed with any malignancy) were both negative for any masses.

Initially, the histopathological examination identified this mass as a germ cell tumor. Based on this diagnosis, the patient received four courses of adjuvant chemotherapy (etoposide 100mg/m^2^, carboplatin 600mg/m^2^, ifosfamide 1800mg/m^2^) according to the International Society of Paediatric Oncology Central Nervous System Germ Cell Tumors II (SIOP CNS GCT II) protocol.

However, after additional histopathological examination and molecular analysis, the final diagnosis of AT/RT was made. After multidisciplinary consultation, the patient underwent radiation therapy: he received a total of 34 Gray (Gy) in 17 fractions to the craniospinal axis and an additional sequential boost of 20 Gy in 10 fractions to the residual tumor. The patient developed leukopenia and thrombocytopenia, which required transfusion therapy and a 3-day course of filgrastim. He otherwise tolerated the radiation therapy regimen well. An adjuvant chemotherapy regimen was started (shortly after radiotherapy) and consisted of vincristine 2mg/m^2^, etoposide 100mg/m^2^, cisplatin 90mg/m^2^, cyclophosphamide 300mg/m^2^, doxorubicin 30mg/m^2^, actinomycin D (dactinomycin) 0.015mg/kg or 1.2mg/m^2^ (depending on chemotherapy cycle) and temozolomid 200mg/m^2^ administered over 14 cycles. Additionally, during each course of chemotherapy, the patient received intrathecal chemotherapy, consisting of methotrexate 15mg/m^2^, cytarabine 60mg/m^2^, prednisolone 30mg/m^2^. The patient well tolerated all these 14 cycles of adjuvant chemotherapy without any major complications. After 7 months since the diagnosis, MRI showed a decrease in the size of the residual tumor. At 21 months’ follow-up, the patient is alive and a further decrease in the size of the residual tumor was shown (**Figures 1G–I**).

### Histopathological examination

As already mentioned, the histological report initially indicated a germ cell tumor. In detail, it described diffuse proliferation of large hyperchromic cells with polymorphic nuclei and the presence of multiple mitoses; some cells had a pronounced pale stained and eosinophilic cytoplasm with the presence of centrally located hypochromic nuclei. Immunohistochemistry showed diffuse immunopositivity for vimentin and focally immunopositivity for Pan Cytokeratin AE1/AE3 and glial fibrillary acidic protein (GFAP).

**Figure 2:**
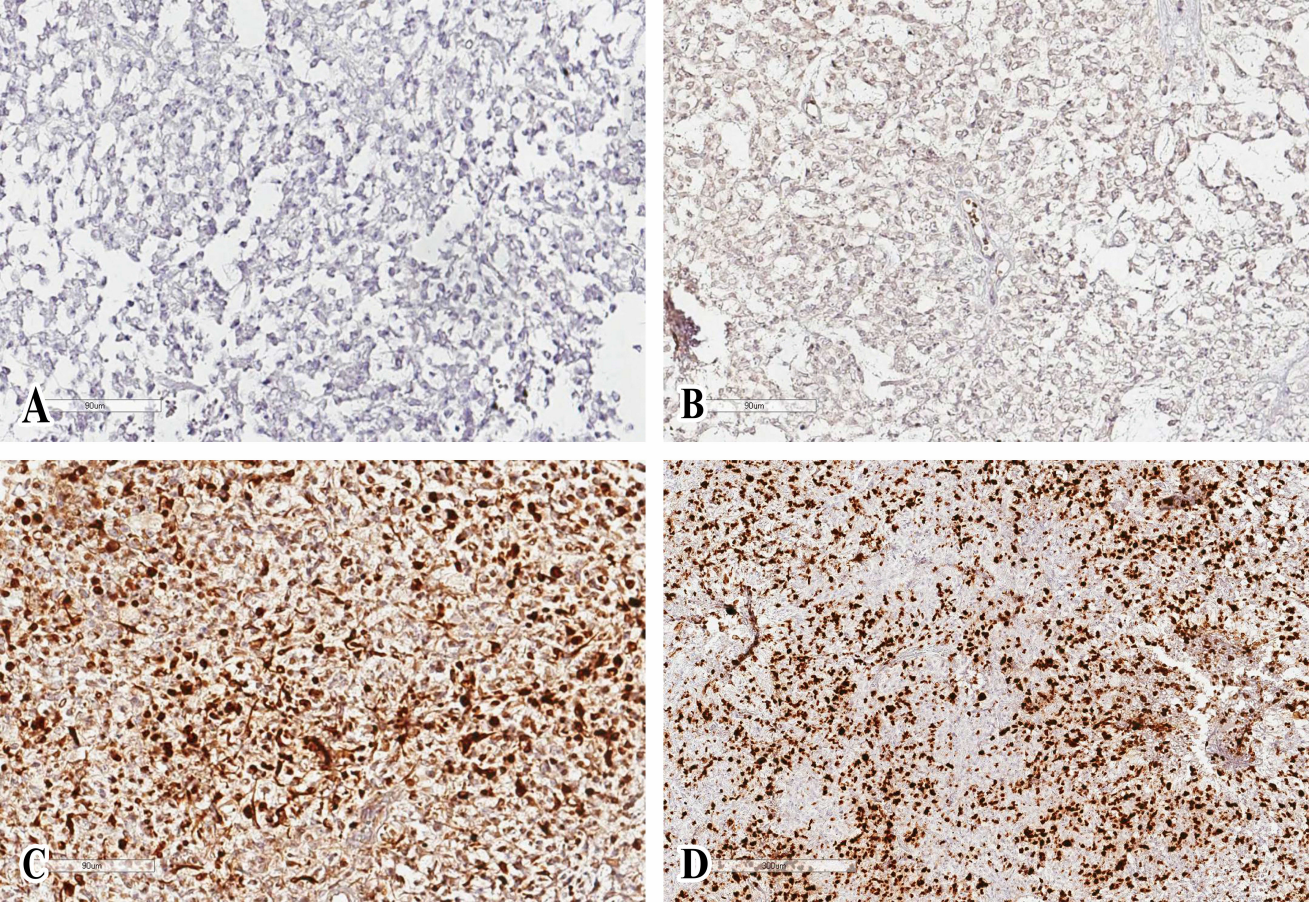
Histologic specimen with features consistent with atypical teratoid/rhabdoid tumor. Glial fibrillary acidic protein **(A)** and epithelial membrane antigen **(B)** immunochemistry demonstrates diffuse positivity. Cytokeratin AE1/AE3 **(C)** was not positive in tumor cells. The proliferative activity of tumor cells is high, the Ki-67 index is 40-50% **(D)**.

After a second histopathological examination and molecular analysis, AT/RT was diagnosed: it described tumor cells with pleomorphic nuclei characterized by euchromatic chromatin, and scarce eosinophilic cytoplasm. There were areas showing tumor cells with typical rhabdoid appearance characterized by abundant eosinophilic cytoplasm and relatively prominent nucleoli. Mitotic activity of cells was elevated, and areas of necrosis and numerous apoptotic bodies were detectable. Immunohistochemistry showed the loss of expression of nuclear integrase interactor 1 (INI1). Furthermore, tumor cells demonstrated strongly immunopositivity for vimentin and microtubule-associated protein two, and focal immunopositivity for epithelial membrane antigen and GFAP (**Figure 2**). The full immunohistochemical panel is summarized in **Supplement Table 1**.

Additionally, molecular analysis by Illumina EPIC Array was performed in order to obtain the genome-wide DNA methylation profile of the tumor cells. The DNA methylation profile was indicative of the presence of a tumor from methylation class «Atypical teratoid/rhabdoid tumor» (classifier score 0.98; the cutoff for classification 0.9, www.molecularneuropathology.org; classifier version V11b4). In addition, a loss of SMARCB1 was detected in a copy number profile based on the EPIC array. Therefore, these additional histopathological examination and molecular analyses confirmed the diagnosis of AT/RT.

## Discussion

AT/RT is a rare and highly malignant CNS embryonal neoplasm: it accounts for <2% of all pediatric CNS tumors. AT/RT is usually diagnosed in infancy and childhood: around 70% and >90% of all cases occur within 1 year and 3 years of age, respectively (8). In terms of tumor site, supratentorial origin is less frequent than infratentorial or spinal occurrence. Recently, Liu YL et al. described their case series including 47 AT/RT patients (study period: 1999-2014): most supra-tentorial tumors were reported in children older than 3 years. This observation is consistent with the previous clinical experiences, as described by Tekautz TM et al. (study period: 1984-2003) and Hilden JM et al. (study period: not specified). Here, we described a pediatric case affected with supra-tentorial AT/RT of the lateral ventricle: only 8 cases were previously described in the medical literature as originating from this specific supra-tentorial site, as shown in **Table 2**, which summarizes our case-based literature review of lateral ventricle AT/RT (6, 8–14). Notably, all the previous cases of lateral ventricle AT/RT were 4 years old or younger, and our case report is the first description of AT/RT arising from the lateral ventricle in an adolescent.

**Table 2:** Literature review of cases of pediatric lateral ventricle Atypical Teratoid/Rhabdoid Tumor (6, 8–14).

№	Case	Age at diagnosis	Gender	CSF spread	Extracranial disease	Surgery	Radiotherapy	Chemotherapy	Relapse	Follow-up after diagnosis
1	Donovan et al., 2006 (8)	3 mo	F	No	No	Two-stage gross total excision	n/a	3 cycles (VCR, CPL, CPO, ETO)	No	48 mo (alive)
2	Meyers et al., 2006 (9)	4 yrs	F	n/a	n/a	Partial excision	n/a	n/a	n/a	n/a
3	Lee et al., 2009 (10)	1.3 yrs	M	No	n/a	Gross total excision	n/a	n/a	n/a	n/a
4	Li et al., 2012 (11)	3 yrs	M	n/a	n/a	Gross total excision	Craniospinal irradiation	n/a	n/a	24 mo (deceased)
5	Darmoul et al., 2015 (12)	2 mo	M	n/a	No	Gross total excision	n/a	1-year (BBSFOP protocol)	No	36 mo (alive)
6	Singh et al., 2016 (6)	4 yrs.	F	n/a	n/a	Gross total excision	n/a	n/a	n/a	n/a
7	Lakhdar et al., 2020 (13)	4 yrs.	M	n/a	No	Gross total excision	Adjuvant (36 Gy/20 fr. + 20 Gy/10 fr.)	4 cycles (VCR, CPL, CPO)	Local (12 mo) Local (21 mo)	23 mo (deceased)
8	Sharma et al., 2020 (14)	4 yrs.	M	Yes	n/a	Gross total excision	n/a	“Adjuvant”	Local (2 mo)	3 mo (deceased)
9	Present case	15 yrs.	M	No	No	Partial excision	Craniospinal (34 Gy/17 fr.) Residual tumor (20 Gy/10 fr.)	14 cycles (VCR, CPL, CPO, ETO, DOXO, ACM-D, temozolomid)	No	21 mo (alive)

F, female; M, male; n/a, information not available; fr., fractions; VCR, vincristine; CPL, cisplatin; CPO, cyclophosphamide; ETO, etoposide; DOXO, doxorubicin; ACM-D, actinomycin-D; yrs., years; mo, months.

The main clinical characteristics and the general medical management of these 9 cases of lateral ventricle AT/RT in children (including ours) are shown in **Table 2**. Overall, all patients underwent surgical intervention to remove the tumor mass, three of them received radiotherapy, and five were treated with adjuvant chemotherapy (vincristine, cisplatin, etoposide, and cyclophosphamide: n=2; vincristine, cisplatin, and cyclophosphamide: n=1; chemotherapy according to the BBSFOP protocol (15): n=1; not specified: n=4). In terms of outcome, among the patients with available follow-up information, three patients died at 3, 23 and 24 months after the diagnosis, whereas three patients (including ours) were still alive at 21, 36, 48 months after the diagnosis.

Recently, Mousa A et al. described their case series including 43 AT/RT patients (study period: 1996-2013): they neither showed significant difference in survival between male and female patients nor between supratentorial and infratentorial localization. The median overall survival for the entire cohort of this study was 16.9 months. Overall, their study confirmed the importance of multimodal treatment, since patients treated this way showed a significantly longer median overall survival compared to patients who received only partial resection and/or limited chemotherapy/radiotherapy (16). These findings are similar to those emerging from the study by Wang RF et al. (study period: 2010-2015), which suggested that older age, gross total surgical resection, and radiation and/or chemotherapy were positive factors for survival (17).

Compared to other CNS sites of tumor development, lateral ventricle AT/RT is more frequently associated to clinical manifestations related to increased intracranial pressure. Neurological symptoms, such as ataxia, vertigo, strabismus, and seizures, seem to be less frequent or evident than in children with lateral ventricle AT/RT compared to the other CNS locations or, in general, other CNS tumors (6). Notably, patients with AT/RT usually have a short clinical history ranging from days to weeks with an average duration of 0.75 months (18), whereas our patient had at least a 2-month clinical history before the CNS mass was investigated and detected, which may be explained by the ventricular location of the tumor. However, the lack of readily available MRI equipment in the regional hospitals may have also affected this late diagnosis.

The radiological features of AT/RT are nonspecific, although large infratentorial tumor with signs of bleeding and meningeal dissemination could be highly suggestive (11). Therefore, the supratentorial location and, in detail, the lateral ventricle origin site has further hampered the exact diagnosis in our case, even though some imaging aspects could be consistent with AT/RT. Indeed, at the MRI AT/RT tumor appears iso- or hypo-intense on the T1-weighted sequence and heterogeneous on the T2-weighted sequence, with a combination of hypointense to hyperintense regions, indicating a site of necrosis, hemorrhage, and cystic changes (8, 13). Our patient’s MRI report was consistent with this description. These MRI characteristics might help to determine the differential diagnosis of common intraventricular tumors (such as primitive neuroectodermal tumor, ependymoma, choroid plexus tumors) and craniopharyngioma (8); however, the histopathological examination is fundamental to confirm the diagnosis.

Actually, a complete panel of immunohistochemical analyses and, possibly, genetic tests, are important to achieve the correct diagnosis, as demonstrated by our case report. Indeed, several tumors can be characterized by cells showing rhabdoid morphology with eccentric nuclei displaced by abundant eosinophilic cytoplasm (1). These aspects can be found in medulloblastoma, primitive neuroectodermal tumor, high-grade glioma, melanoma, rhabdoid meningioma, or a variety of metastatic tumors (12). Therefore, the immunohistochemical examination is essential for distinguishing AT/RT from other CNS neoplasms, even though some aspects may vary depending on the cellular composition of the tumor. Rhabdoid cells usually demonstrate clear expression of vimentin, epithelial membrane antigen, and smooth muscle actin. Expression of glial fibrillary acidic protein, neurofilament protein, synaptophysin, and keratin may be variably positive (12).

The most definitive evidence supporting a final diagnosis of AT/RT is the genetic test demonstrating the inactivation or deletion of SMARCB1 and loss of expression of INI1(as it was in our clinical case); the expression loss of BRG1 protein and SMARCA4 mutations can be found less frequently (19–21). SMARCB1 gene is located at the locus of chromosome 22q11.2, and is a core component of the SWItch/Sucrose Non-Fermentable (SWI/SNF) chromatin-remodeling complex. It carries a crucial role in tumor suppression and has been implicated in the pathogenesis of several malignancies (22). Immunohistochemical staining for loss of INI1 expression is considered an integrated approach for diagnosing AT/RT in a consistent histopathological setting, according to the World Health Organization classification criteria of CNS tumors (21).

Modern advances in molecular methods (including genomic, transcriptomic, and epigenomic analyses) to investigate tumors have definitely facilitated and improved the identification of rare, difficult-to-diagnose tumors in the last few years. However, these methods are readily available only to the most advanced and specialized centers in developed countries, whereas many difficulties exist in developing countries, even in pediatric referral center of national relevance, like ours. Limited access to these resources can increase the risk of misdiagnosis, unfortunately. The Austrian Brain Tumor Registry found that 52.6% of AT/RTs diagnosed between 1996 and 2006 were inaccurately diagnosed as other tumor types, most commonly medulloblastoma (2). Two retrospective case series studies by the British Columbia’ Children’s Hospital (1986–2006) and the Institute of Neurology in Vienna, Austria (1964–2005) also found numerous AT/RT cases upon IHC analysis, which where misdiagnosed as other embryonal tumor types (23, 24).

The problem of the limited access to modern methods of tumor diagnostics in developing countries may be partially solved through a cooperation with major cancer academic centers in developed countries. For example, at the moment, our oncology department is collaborating with oncology centers in Russia and in the USA. Such collaborations allow oncological centers in developing countries to get consultation and second opinion from more experienced specialists (in addition to having access to the molecular analyses) in order to better determine the therapeutic strategy to manage patients with rare tumors. In general, a timely diagnosis is very important in CNS tumors and, perhaps, even more in rare tumors (such as AT/RT), for which no standard treatment has been consolidated, yet (25). A multimodal approach including gross resection combined with radiotherapy and/or high-dose chemotherapy may improve the prognosis of AT/RT, but timely diagnosis and early start of the appropriate treatment remain an essential condition to increase the life expectancy and to reduce the iatrogenic complications. Indeed, some studies demonstrated a significant improvement in the overall survival rate after gross total resection compared with subtotal/near-total resection (26, 27).

## Conclusion

Pediatric AT/RT is a rare CNS malignancy. Supratentorial and, in detail, latero-ventricular origin site is extremely uncommon, especially in adolescents. MRI findings are useful to restrict the differential diagnosis to specific CNS malignancies, but the histopathological examination, including a full immunohistochemistry panel, is essential to correctly diagnose AT/RT, especially if the tumor site is unusual. Actually, molecular testing can finally confirm AT/RT diagnosis, allowing the patient to receive the precise diagnosis and, thus, a timely and appropriate treatment, which is the main factor potentially able to improve such a serious prognosis. Finally, implementing a system supporting pathological and molecular analyses for developing countries and, in general, for non-academic centers is of primary importance to timely diagnose and treat rare tumors, such as AT/RT.

## Data availability statement

The original contributions presented in the study are included in the article/**Supplementary Material**. Further inquiries can be directed to the corresponding author.

## Ethics statement

Written informed consent was obtained from the minor(s)’ legal guardian/next of kin for the publication of any potentially identifiable images or data included in this article.

## Author contributions

AK, KS, AS, and DN conceived this publication. AK, KS, and AS collected the clinical data. BI provided the histopathological data. AK and DP wrote the original draft of the manuscript. DP, KS, and DN reviewed the manuscript. All authors contributed to the article and approved the submitted version.

## Acknowledgments

The authors thank the Department of Science and Education of the University Medical Center (UMC) for supporting the publication of this manuscript on the journal.

## Conflict of interest

The authors declare that the research was conducted in the absence of any commercial or financial relationships that could be construed as a potential conflict of interest.

## Publisher’s note

All claims expressed in this article are solely those of the authors and do not necessarily represent those of their affiliated organizations, or those of the publisher, the editors and the reviewers. Any product that may be evaluated in this article, or claim that may be made by its manufacturer, is not guaranteed or endorsed by the publisher.
