# Supplementary material for: Case report: Atypical teratoid/rhabdoid tumor of the lateral ventricle in a male adolescent (case-based review and diagnostic challenges in developing countries)
Source: Front Oncol. 2022 Oct 6;12:985862. doi: 10.3389/fonc.2022.985862 (PMC9582653; doi:10.3389/fonc.2022.985862)
Supplement: Supplementary file 1 [file Table_1.docx]

**Supplementary Table 1**

**Suppl. Table 1. Immuno-histochemical panel and characteristics.**

| Vimentin | Strongly positive in tumor cells |
| --- | --- |
| Integrase Interactor 1/SMARCB1 | Nuclear loss in tumor cells, retained in stromal cell nuclei |
| Brahma-related gene 1/SMARCA4 | Nuclear immunoreactivity in tumor cells |
| Ki67 index | 10-15% |
| Cytokeratin AE1/AE3 | Not positive in tumor cells |
| Epithelial membrane antigen | Focally positive in multiple tumor cells and nests of tumor cells |
| p53 | Nuclear accumulation in 20% of the tumor cells |
| Desmin | Probably unspecific granular staining of the tumor cells |
| Glial fibrillary acidic protein | Positive in only a small amount of tumor cells |
| Microtubule-associated protein 2 | Strongly positive in tumor cells |
| Smooth muscle actin | Probably unspecific granular staining of the tumor cells |
| CD45 | Multiple diffusely located leukocytes |
| Oct4 | No nuclear positivity in tumor cells |
| Beta-human chorionic gonadotropin | Not positive in tumor cells |
| Alpha-fetoprotein | Not positive in tumor cells |
| Placental alkaline phosphatase | Not positive in tumor cells |
| CD117 | Probably unspecific granular staining of tumor cells |
